# Mitofusin 1 is degraded at G_2_/M phase through ubiquitylation by MARCH5

**DOI:** 10.1186/1747-1028-7-25

**Published:** 2012-12-20

**Authors:** Yong-Yea Park, Hyeseong Cho

**Affiliations:** 1 Department of Biochemistry, Ajou University School of Medicine, Suwon, South Korea; 2 Department of Biochemistry, Ajou University School of Medicine, Graduate School of Molecular Science and Technology, Ajou University, 5 Wonchoen-dong, Yeongtong-gu, Suwon, 443-721, South Korea

**Keywords:** MARCH5, Mfn1, G_2_/M, Mitochondrial fragmentation

## Abstract

**Background:**

Mitochondria exhibit a dynamic morphology in cells and their biogenesis and function are integrated with the nuclear cell cycle. In mitotic cells, the filamentous network structure of mitochondria takes on a fragmented form. To date, however, whether mitochondrial fusion activity is regulated in mitosis has yet to be elucidated.

**Findings:**

Here, we report that mitochondria were found to be fragmented in G_2_ phase prior to mitotic entry. Mitofusin 1 (Mfn1), a mitochondrial fusion protein, interacted with cyclin B1, and their interactions became stronger in G_2_/M phase. In addition, MARCH5, a mitochondrial E3 ubiquitin ligase, reduced Mfn1 levels and the MARCH5-mediated Mfn1 ubiquitylation were enhanced in G_2_/M phase.

**Conclusions:**

Mfn1 is degraded through the MARCH5-mediated ubiquitylation in G_2_/M phase and the cell cycle-dependent degradation of Mfn1 could be facilitated by interaction with cyclin B1/Cdk1 complexes.

## Findings

Mitochondria are multifunctional organelles that play essential roles in many cellular functions, including respiration, ATP production, and calcium buffering [1,2]. In most cell types, mitochondria form a tubular network structure whose morphology changes dynamically through a controlled balance of fusion and fission events. Mitofusins (Mfn1/2) and Opa1 (optic atrophy 1) are essential for the fusion of mitochondrial outer and inner membranes, respectively. Drp1 (dynamin related protein 1) has pivotal roles in fission events through association with Fis1 (fission 1) or Mff [3,4]. An imbalance in mitochondrial dynamics affects cellular physiology. For example, abnormally elongated mitochondria, caused by depletion of Fis1 or MARCH5, induce cellular senescence [5,6]. Moreover, extensive mitochondrial fragmentation is observed in apoptotic cells, whereas cells with elongated mitochondria are more resistant to apoptotic stimuli [7,8].

Mitochondrial biogenesis and function are integrated with the nuclear cell cycle. Diminished production of ATP caused by mutations in the mitochondrial protein, *tenured* (tend), which encodes a cytochrome oxidase subunit Va, causes G_1_/S cell-cycle arrest during larval growth in *Drosophila*[9]. Mitochondrial function is also affected by p53 [10]. Additionally, mitochondria are known to show dynamic morphology during cell-cycle stages [11-13]. Cyclin D1, a G_1_/S-phase controlling protein, represses mitochondrial function and size [10,14]. During G_1_/S phase, mitochondria form a hyper-fused giant network that regulates cyclin E accumulation [15]; this thin, filamentous structure radiates from the perinuclear region to the periphery and its formation increases mitochondrial mass [11]. Subsequent cell-cycle events during G_2_/M phase act as a checkpoint against DNA damage and ensure entry into mitosis where chromosomes are partitioned equally to two daughter cells. Other organelles and cellular components must also be segregated between the two daughter cells during mitosis. It is reported that mitochondrial fragmentation occurs early in mitosis (prophase) and requires phosphorylation of Drp1 by Cdk1/cyclin B1 [11,16]. It is also shown that mitochondria fully develop their mitochondrial membrane potential at G_2_/M [17]. Despite the connection between mitochondrial function/dynamics and cell-cycle progression, relatively little is known about the underlying mechanism. This is especially true with respect to the function of mitochondrial fusion proteins in the mitotic fragmentation of mitochondria.

Here, we attempted to address whether mitochondrial fusion activity is also regulated during G_2_/M phase. We recognized that the mitochondrial fusion protein, Mfn1, contains a putative target sequence for phosphorylation by the cyclin B1/Cdk1 complex and in fact found that Mfn1 interacted with cyclin B1. In G_2_/M phase, ubiquitylation of Mfn1 by MARCH5 is increased and Mfn1 levels are reduced through proteasome-mediated degradation. Thus, cell cycle-dependent degradation of Mfn1 could be facilitated by interaction with cyclin B1/Cdk1 complexes.

### Mfn1 interacts with Cdk1/cyclin B1

**Figure 1:**
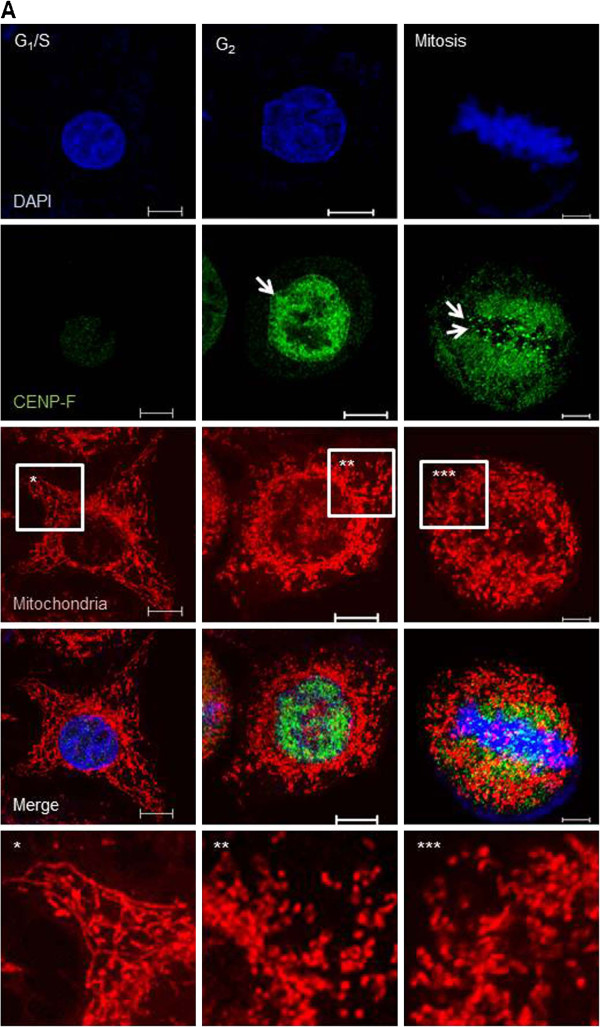
***Formation of fragmented mitochondria at G***_***2 ***_***phase.*** (**A**) Cells were synchronized and released at the indicated phase using the DTB method. Images were analyzed by confocal microcopy. Scale bars = 10 μm.

To investigate mitochondrial morphological changes during cell-cycle progression in detail, we synchronized HeLa cells at G_1_/S using the double-thymidine block (DTB) method, after which mitochondria were stained with MitoTracker Red and analyzed by confocal microscopy. CENP-F, which predominantly localizes to the nuclear matrix at G_2_ phase and moves to the nuclear membrane and kinetochores during late G_2_ phase and mitosis, respectively [18], was used as a cell-cycle marker. In G_1_/S-arrested cells, mitochondria formed an interconnected network structure (Figure 1, left). However, G_2_-phase cells showed fragmented mitochondria (Figure 1, middle), suggesting that mitochondria undergo fragmentation before mitotic entry. In mitotic cells, mitochondria fragmented remained small and punctate form (Figure 1, right). These changes in mitochondrial morphological features during the cell cycle are mostly consistent with previous studies [11,12,17,19]; however, the mechanism underlying these mitochondrial dynamics have been partly understood.

**Figure 2:**
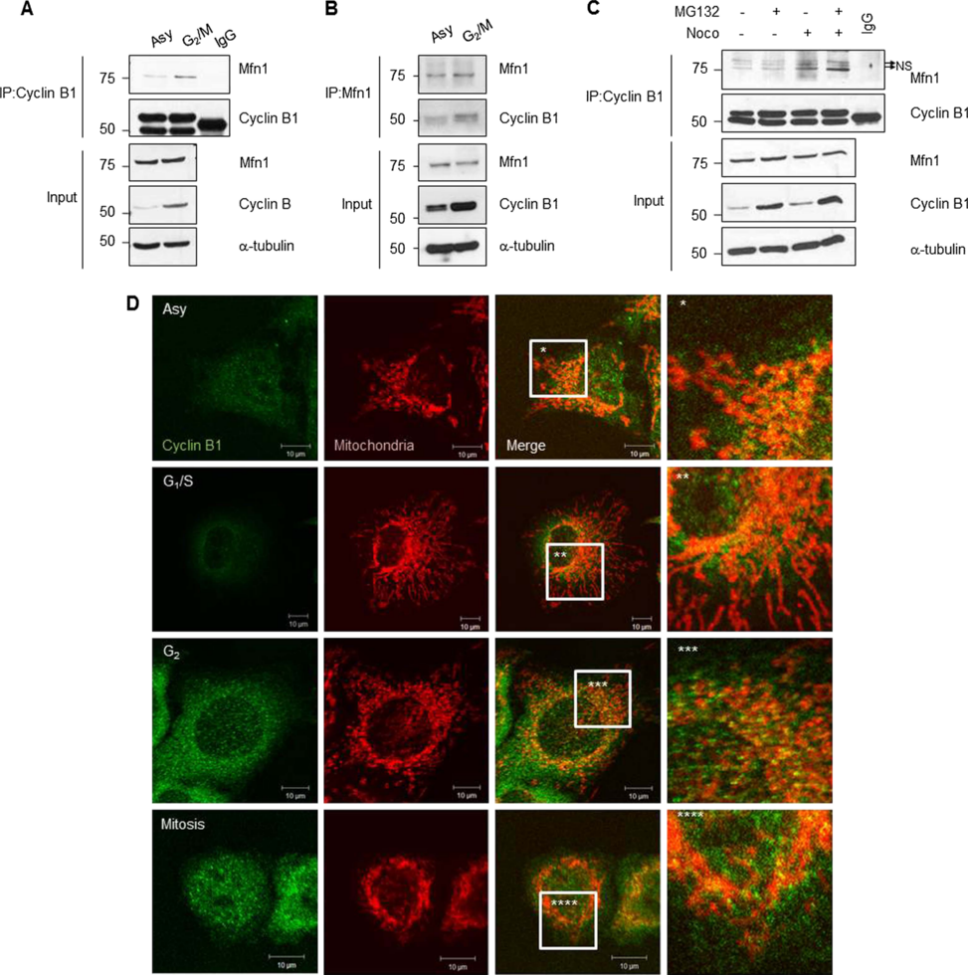
***Mfn 1 interacts with Cdk1/cyclin B1.*** (**A**) G_2_/M-phase cells were obtained by harvesting cells after nocodazole treatment. Lysates were immunoprecipitated with an anti-cyclin B1 antibody, followed by immunoblotting with the indicated antibodies. (**B**) G_2_/M-phase cells were obtained by taxol treatment for 12 h. The association to cyclin B1 was analyzed by immunoblotting, after lysates were immunoprecipitated with an anti-Mfn1 antibody. (**C**) Cells were treated with MG132 with or without nocodazole. Lysates were immunoprecipitated with an anti-cyclin B1 antibody, followed by immunoblotting with the indicated antibodies. Arrows (NS, upper two bands) indicate non-specific binding. (**D**) Cells were synchronized at G1/S, G_2_ or mitotic phase by treatment with thymidine, roscovitine (Ros, 100 μM) for 5 h at 7 h after DTB release or nocodazole for 2 h at R8 after DTB release, respectively. Mitochondrial morphology was observed by staining with MitoTracker Red. Images were analyzed by confocal microcopy. Scale bars = 10 μm.

It is known that the mitochondrial fission factor Drp1 is phosphorylated by Cdk1/cyclin B1 and phosphorylated-Drp1 induces mitochondrial fragmentation, which is important in G_2_/M transition [16]. However, despite a general recognition that mitochondrial morphology is controlled through a balancing of fusion and fission, the relationship between mitotic fragmentation and mitochondrial fusion has received little research attention. We thus attempted to identify the mechanism regulating the fusion event at the G_2_/M transition. During this transition, multiple possible post-translational modifications of fusion factors might take place. Accordingly, we hypothesized that mitochondrial fusion factors are regulated during cell-cycle progression. We recognized that Mfn1 contains one potential recognition site (at serine 6) of cyclin B1/Cdk1, a serine/threonine kinase that recognizes the consensus motif [S/T]PX[K/R] [20]. Therefore, we examined whether Mfn1 interacts with cyclin B1 using co-immunoprecipitation assay. To collect cells in G_2_/M phase, cells were collected after treatment with nocodazole or taxol for 12 h. Co-immunoprecipitates using anti-cyclin B1 antibody revealed that Mfn1 interacted with cyclin B1 and the levels of cyclin B increase and there is more of it bound to Mfn1 in G_2_/M phase than those in asynchnously (Asy) growing cells (Figure 2A, C). Reciprocal co-immunoprecipitation with anti-Mfn1 antibody also showed the interaction of Mfn1 to cyclinB/Cdk1 complex (Figure 2B). Since Mfn1 is localized to the mitochondrial outer membrane, we next examined whether cyclin B1 is also localized to mitochondria. Immunofluorescence staining showed an accumulation of cyclin B1 in G_2_/M phase (Figure 2D, left). Co-immunostaining with mitochondria displayed the speckles of cyclin B1 (green) overlaid with mitochondria (Figure 2D, right) in G_2_/M phase. Thus, the data suggest that Mfn1 may serve as a substrate of the Cdk1/cyclin B1 complex in G_2_/M phase. In addition, the interaction between Mfn1 and Cdk1/cyclin B1 was increased in the presence of MG132, a proteasome inhibitor (Figure 2C), also suggesting that the binding of Mfn1 to the cyclin B1/Cdk1 complex stimulate the degradation of Mfn1 through a proteasome-dependent pathway.

### Mfn1 levels are regulated by MARCH5-mediated ubiquitylation in G_2_/M phase

**Figure 3:**
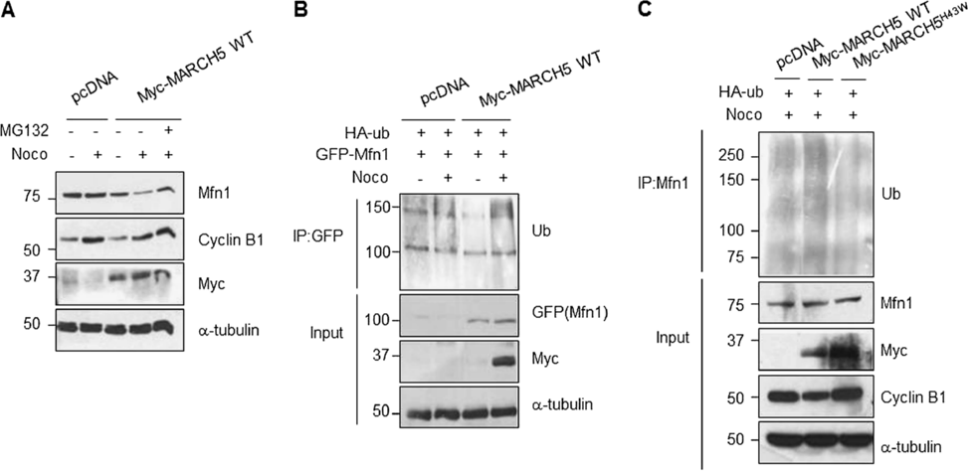
***Mfn1 is ubiquitylated by MARCH5 at G***_***2 ***_***phase.*** (**A**) Cells were transfected with pcDNA or Myc-MARCH5 WT, and exposed to nocodazole. Myc-MARCH5 WT-expressing cells were incubated with MG132. Lysates were analyzed by immunoblotting with the indicated antibodies. (**B**) Cells were co-transfected with HA-ub. GFP-Mfn1 with pcDNA or Myc-MARCH5 WT. G_2_/M-arrested cells were obtained by treatment with nocodazole and then treated with MG132. Lysates were immunoprecipitated with an anti-GFP antibody for ubiquitylation assays. GFP-Mfn1 ubiquitylation levels were evaluated with an anti-ub antibody. (**C**) Cells were co-transfected with HA-ub and pcDNA, Mcy-MARCH5 WT or Myc-MARCH5^H43W^, after which cells were synchronized at G_2_/M by treatment with nocodazole with MG132. The ubiquitylation levels of endogenous Mfn1 were assayed by immunoblotting.

MARCH5 is a mitochondrial E3-ubiquitin ligase that localizes to the mitochondrial outer-membrane and ubiquitylates proteins that control mitochondrial dynamics [6,21-23]. As we reported recently, Mfn1 is a novel substrate of MARCH5. Though Mfn1 shows high similarity of amino acid sequence with Mfn2, the expression levels of Mfn2 were not affected by MARCH5 [6]. Thus, we next investigated whether Mfn1 is regulated by MARCH5 in a cell cycle-dependent manner. We observed that Mfn1 levels were decreased in HeLa cells overexpressing MARCH5 (Figure 3A). It is of note that Mfn1 levels were even more reduced in cells treated with nocodazole (Figure 3A, compare lanes 3 and 4), indicating that the MARCH5-mediated reduction of Mfn1 levels is accelerated in G_2_/M phase. Moreover, the decreased level of Mfn1 was recovered by blocking the proteasome-mediated degradation with MG132 (Figure 3A, Lane 6). Taken together, our results suggest that degradation of Mfn1 at the G_2_/M phase is regulated by MARCH5.

Next, to verify that MARCH5 ubiquitylates Mfn1 in G_2_/M phase, we performed ubiquitylation assays in HeLa cells co-transfected with GFP-Mfn1 and Myc-MARCH5, and synchronized at G_2_/M by treatment with nocodazole. Interestingly, G_2_/M-phase cells expressing wild-type MARCH5 (Myc-MARCH5 WT) showed higher levels of Mfn1 ubiquitylation, indicating that Mfn1 is extensively ubiquitylated at G_2_/M by MARCH5 (Figure 3B). Next, we clarified whether the ubiquitylation of Mfn1 is dependent on the RING domain of MARCH5 [6]. After cells were co-transfected with HA-tagged ubiquitin and Myc-MARCH5 WT or Myc-MARCH5^H43W^, containing an inactivating mutation in the RING domain, G_2_/M-phase cells were obtained using nocodazole treatment. Lysates were immunoprecipitated with an anti-Mfn1 antibody and ubiquitylation levels were assessed by immunoblotting. Extensively poly-ubiquitylated Mfn1 was observed in G_2_/M-phase cells. Notably, the ubiquitylation of Mfn1 was reduced in Myc-MARCH5^H43W^, indicating that MARCH5 regulates the ubiquitylation of Mfn1 at the G_2_/M phase (Figure 3C).

In conclusion, we here postulate a new cell cycle-dependent regulatory mechanism for Mfn1. Mfn1 is ubiquitylated by MARCH5 at G_2_/M phase and degraded in a proteasome-dependent manner. Furthermore, there is likely that the MARCH5-mediated Mfn1 degradation in G_2_/M phase can be modulated by Cdk1/cyclin B1 kinase.

## Materials and methods

### Cell culture, cell synchronization, and transfection

HeLa cells were maintained in Dulbecco’s modified Eagle’s medium (DMEM; Invitrogen) supplemented with 10% heat-inactivated fetal bovine serum and 1% antibiotic-antimycotic at 37°C in a humidified 5% CO_2_ atmosphere. Plasmid DNA were transfected to the cells using polyethylenimine (PEI; Polysciences) as previously described [6]. HeLa cells were synchronized at the G_1_/S boundary using the DTB method. Briefly, cells were incubated with 2 mM thymidine for 20 h, followed by release in thymidine-free medium for 8 h. The cells were incubated one more time with 2 mM thymidine for 16 h. G_2_/M-arrested cells were isolated by treating with 100 ng/ml of nocodazole for 12 h before harvesting.

### Plasmid construction

The mammalian MARCH5-YFP expression plasmid has been described previously [6]. The Myc-MARCH5 expression plasmid was created by sub-cloning MARCH5-YFP with 3x-Myc into pcDNA3.1(−)MARCH5 was digested with *Xho*I and *Bam*H1, 3x-Myc was digested with *Nhe*I and *Xho*I, and the two fragments were ligated into pcDNA 3.1(−) that had been digested with *Nhe*1 and *Bam*H1.

### Immunocytochemistry and confocal microscopy

HeLa cells were seeded on coverslips and synchronized using the DTB method or by treatment of 100 ng/ml nocodazole. The synchronized cells were then incubated with 125 nM MitoTracker Red (Molecular Probes) for 30 min, washed and fixed with 4% paraformaldehyde solution for 10 min, and then washed three times with phosphate-buffered saline (PBS). Fixed cells were permeabilized by incubating with methanol for 20 min at -20°C. For immunofluorescence staining, cells were blocked with 1% bovine serum albumin in PBS for 1 h at room temperature, followed by incubation first with primary antibodies overnight at 4°C and then with fluorescence-conjugated secondary antibodies. Images were obtained and analyzed using an LSM510 Confocal Microscope (Carl Zeiss).

### Immunoprecipitation and *in vivo* ubiquitylation assay

For immunoprecipitation experiments, HeLa cells were synchronized at G_2_/M phase with 10 ng/ml of nocodazole, and mitotic floating cells and residual adherent cells were harvested separately or collectively. Collected cells were suspended and sonicated in immunoprecipitation (IP) buffer (50 mM HEPES pH 7.5, 150 mM NaCl, 0.1% NP-40, 5 mM EDTA, 1 mM DTT, and protease inhibitor). For cyclin B11 immunoprecipitations, whole-cell lysates containing 500–1500 μg of proteins were immunoprecipitated with 1 μg of anti-cyclin B11 antibody by incubating at 4°C overnight with agitation. Protein-antibody complexes were further incubated with 25 μlof protein A-sepharose beads (GE Healthcare Bio-Science AB) for 1 h and 30 min. After washing beads and protein complexes four times with IP buffer followed by heating in 2X sample buffer, samples were separated by SDS-PAGE and analyzed by immunoblotting. Mfn1 immunoprecipitates were prepared and analyzed by immunoblotting as described for cyclin B11 immunoprecipitates. For *In Vivo* ubiquitinylation assays, HeLa cells synchronized with 10 ng/ml of nocodazole were treated 20 μM MG132 for 4 h before harvesting.

### Immunoblotting and antibodies

Cells were lysed and sonicated in E1A lysis buffer (50 mM HEPES pH 7.5, 150 mM NaCl, 0.1% NP-40, 5 mM EDTA, 1 mM DTT, and protease inhibitor) followed by centrifugation. Supernatants were normalized for protein content and boiled with Laemmli sample buffer. The lysates were separated by SDS-PAGE and analyzed by immunoblotting with anti-Mfn1 (Protein Tech), anti-Cyclin B11, anti-ubiquitin and anti-c-Myc (Santa Cruz Biotechnology) antibodies.

## Abbreviations

Mfn1: Mitofusin1; MARCH5: Membrane-associated ring finger (C3HC4) 5; Drp1: Dynamin related protein 1; Fis1: Fission 1; DTB: Double-thymidine block.

## Competing interests

The authors declare no competing interests.

## Authors’ contributions

Both authors carried out the experiments and approved the manuscript.
